# Long-term outcome of catheter ablation in patients with persistent atrial fibrillation and functional tricuspid regurgitation

**DOI:** 10.3389/fcvm.2025.1600238

**Published:** 2025-11-07

**Authors:** Wenwu Liu, Anlu Wang, Qiuyi Li, Qitong Zhang, Songwen Chen, Yong Wei, Xiaofeng Lu, Xiaoyu Wu, Bin Dai, Shaowen Liu, Genqinq Zhou

**Affiliations:** 1 Department of Cardiology, Shanghai General Hospital of Nanjing Medical University, Shanghai, China; 2 Department of Cardiology, Suzhou TCM Hospital Affiliated to Nanjing University of Chinese Medicine, Suzhou, Jiangsu, China; 3 National Clinical Research Center for Chinese Medicine Cardiology, Xiyuan Hospital, China Academy of Chinese Medical Sciences, Beijing, China; 4 Department of Cardiology, Shanghai General Hospital, Shanghai Jiaotong University School of Medicine, Shanghai, China

**Keywords:** atrial fibrillation, functional tricuspid regurgitation, radiofrequency catheter ablation, recurrence, persistent atrial fibrillation

## Abstract

**Background:**

Atrial fibrillation (AF) and tricuspid regurgitation (TR) frequently coexist and mutually worsen each other. However, the long-term effects of functional tricuspid regurgitation (FTR) on AF recurrence and the improvement of TR after radiofrequency catheter ablation (RFCA) remain unclear.

**Methods:**

This retrospective, single-center observational study involved 1,690 patients with persistent atrial fibrillation (PsAF) who underwent AF ablation between January 2012 and June 2022. 153 paients with significant FTR were propensity matching 153 patients with no or mild FTR based on age, body mass index, and mitral regurgitation (MR) severity. Patients were followed up for at least 1 year. Procedural success was defined as freedom from any atrial tachyarrhythmia (>30 s) after the 3-month blanking period, off antiarrhythmic drugs. Significant FTR was defined as moderate to severe TR. Significant TR improvement was defined as a reduction in TR severity by ≥2 grades from pre- to post-ablation.

**Results:**

Among the observational cohort, 153 patients (9.1%) had significant FTR, the severity of which correlated with female sex, AF duration, atrial/ventricular remodeling, and NT-proBNP levels. During the 12-month follow-up, the procedural success rate was 67.6% (207/306). RFCA significantly reduced the proportion of RA and RV enlargement (*P* < 0.001), and improved the severity of FTR (*P* < 0.001). Logistic regression analyses showed that AF recurrence [odds ratio (OR) 18.244, 95% CI 7.500–52.427, *P* *<* 0.001]) was the strongest independent risk factor for non-significant TR improvement after ablation. After a mean follow-up of 5.3 ± 3.7 years, the overall procedural success rate was 47.4% (145/306). The overall success rate was significantly lower in patients with significant FTR compared to those with no or mild FTR (37.3% VS 57.5%, *P* < 0.001). A comparable difference was observed between ventricular FTR and no or atrial FTR (27.1% VS 53.4%, *P* < 0.001).

**Conclusions:**

Significant FTR was an independent predictor of AF recurrence in patients with PsAF undergoing RFCA. The long-term success was poor in the subgroups of significant FTR and those with ventricular FTR. Furthermore, successful RFCA was associated with significantly improved FTR in patients with PsAF.

## 1 Introduction

Atrial fibrillation (AF) is commonly encountered in clinical practice, the global prevalence of AF in adults is estimated to be between 2% and 4%, and its incidence increases with age (1). AF not only impacts patients' quality of life but also gives rise to various severe complications, such as stroke and heart failure, leading to substantial economic and social burdens (2). Functional tricuspid regurgitation (FTR), also known as secondary tricuspid regurgitation (TR), is defined as leakage of the tricuspid valve during systole in the presence of structurally normal leaflets and chordae. FTR accounts for more than 80% of all TR cases and is often overlooked due to subtle symptoms in the early stage, and patients with moderate or more severe FTR are related with an increased risk of heart failure and mortality (3). The incidence of FTR in the general population is 0.55% and increases with age, in patients over 75 years old, the incidence of FTR is approximately 4% (4). According to recent literature (5–7), there are two distinct etiologies of FTR. FTR with right ventricular (RV) enlargement or dysfunction is commonly referred to as ventricular FTR (vFTR), which is typical in patients with pulmonary artery (PA) hypertension. On the other hand, patients with atrial FTR (aFTR), which was formerly identified as idiopathic or isolated TR, present with a normal RV and preserved RV systolic function.

Recent clinical evidences have established AF as an independent risk factor for FTR progression. This bidirectional relationship manifests clinically as frequent co-occurrence and mutual aggravation of both conditions (8, 9). A previous study discovered that FTR was a predictive factor for AF recurrence after radiofrequency catheter ablation (RFCA) (10). However, the long-term follow-up results of RFCA in persistent AF (PsAF) patients with FTR and different FTR subtypes are relatively scarce, and the effect of RFCA on the improvement of TR needs further investigation. This study aimed to evaluate long-term outcomes of RFCA in PsAF patients with FTR, identify predictors of AF recurrence, and assess the therapeutic impact on FTR.

## 2 Methods

### 2.1 Study design

The clinical retrospective investigation was conducted at Shanghai General Hospital. The inclusion criteria were as follows: (1) PsAF lasting more than 7 days, as defined by the European Society of Cardiology guidelines (11); (2) RFCA procedure of AF was performed for the first time. FTR was defined as valvular regurgitation in the presence of structurally normal leaflets and chordae, with or without right atrial/ventricular enlargement and ventricular dysfunction. Significant FTR was defined as at least moderate TR grade. Patients who had previous ablation for AF, as well as those with pacemakers, prosthetic heart valves, tricuspid valve prolapse, endocarditis, tumors, or congenital heart diseases, were excluded from this study. All patients provided written informed consent prior to the procedure. The study protocol was approved by the institutional ethics review board.

### 2.2 Study population

**Figure 1:**
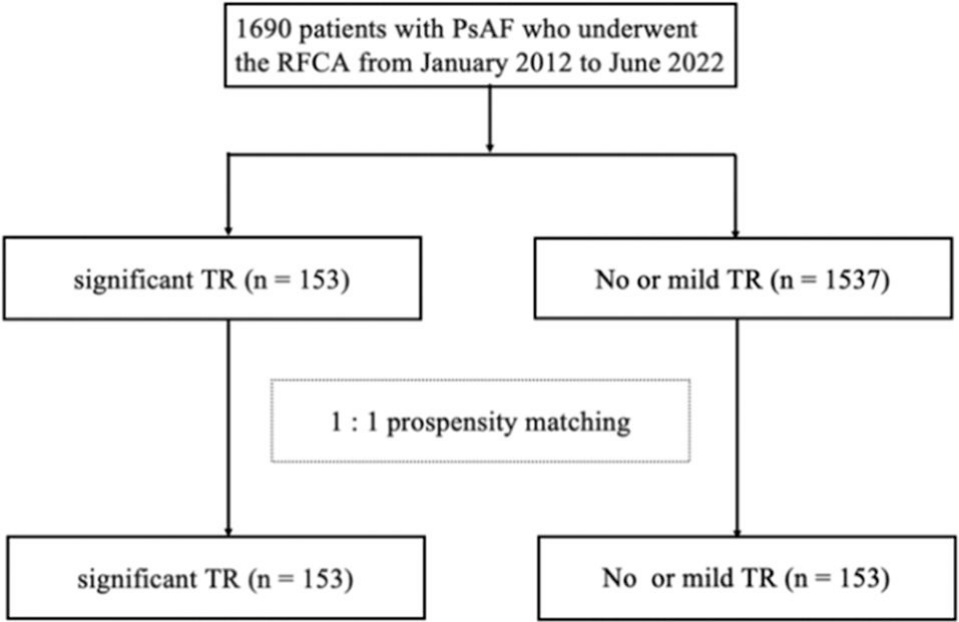
Flow chart of subject selection. AF, atrial fibrillation; RFCA, radiofrequency catheter ablation; PsAF, persistent atrial fibrillation; TR, tricuspid regurgitation.

We conducted a retrospective analysis of 1,690 patients with PsAF who underwent RFCA at Shanghai General Hospital between January 2012 and June 2022. Of the 1,690 patients with PsAF, ultimately, 153 patients with significant FTR and 153 patients propensity-matched controls for age, body mass index (BMI) and presence of significant MR who underwent first-time ablation were included in the study (Figure 1).

### 2.3 Echocardiography

All patients underwent transthoracic echocardiography and transesophageal echocardiography examination within 3 days before ablation procedure. The cardiac structure measurements were obtained using two-dimensional ultrasound and color Doppler ultrasound in various views, including parasternal short-axis and long-axis views, as well as apical two-chamber and four-chamber views. PA pressure, defined as the systolic pulmonary artery pressure, was determined by measuring the maximum tricuspid regurgitation velocity and adding an estimated central venous pressure. The area of functional mitral regurgitation (MR) was directly delineated from the apical four-chamber view, two-chamber view, and apical long-axis view. The morphology and area of TR were observed using color doppler imaging from multiple views. The evaluations of TR were in accordance with the guidelines of the American Society of Echocardiography (12). TR area less than 5 cm^2^ was classified as mild, 5–10 cm^2^ as moderate-severe, and greater than 10 cm^2^ as severe. TR severity was categorized into 5 grades based on the severity of TR: none (0), mild (1), moderate (2), moderate-to-severe (3), and severe (4). MR severity grading followed the same categorical scale. In this study, Significant FTR was defined as moderate to severe TR (TR grade ≥2). FTR accompanied by RV enlargement or dysfunction was defined as vFTR, whereas aFTR was characterized by the absence of these RV abnormalities. Significant TR improvement was defined as a decrease of at least two grades in TR (referring to 5-grade severity) following ablation compared to the pre-procedure evaluation.

### 2.4 Radiofrequency catheter ablation

All patients received oral anticoagulation for a minimum of 3 weeks before the procedure. New oral anticoagulants were discontinued on the procedural day. However, oral warfarin was maintained to ensure that the international normalized ratio (INR) level remained between 2.0 and 3.0.

A 10-electrode catheter was inserted into the coronary sinus through either the subclavian vein or femoral vein. Following trans-septal puncture, intravenous unfractionated heparin (initial dose of 80–120 U/kg) was administered to maintain the activated clotting time level between 300 and 350 s. Analgesia and anesthesia were provided using midazolam and fentanyl.

A Lasso or PentaRay catheter (Biosense Webster, Inc.), along with an irrigated radiofrequency ablation catheter (Thermo-Cool, Thermo-Cool Smart-Touch, or Thermo-Cool Smart-Touch SF, Biosense Webster, Inc.), were advanced into the left atrial (LA) guided by a three-dimensional navigation system (CARTO or CARTO 3, Biosense Webster, Inc.).

The electrophysiological study was performed using either a Lasso catheter (Biosense Webster, Inc.) or PentaRay catheter (Biosense Webster, Inc.) for mapping, along with an irrigated radiofrequency ablation catheter (ThermoCool, ThermoCool SmartTouch, or ThermoCool SmartTouch SF; Biosense Webster, Inc.). All catheters were advanced into the LA guided of a three-dimensional navigation system (CARTO or CARTO 3, Biosense Webster, Inc.). Point-by-point ablation technique was employed for ablation, by using the power control mode. Each radiofrequency energy delivery lasted for 20–60 s, with power output set at 35–45W in the atrium, and 20–30W in the coronary sinus and during superior vena cava isolation, and the flow rate was maintained between 8 and 30 ml/min.

After March 2014, the force-sensing catheters (Biosense Webster, Inc.) were systemically utilized, with the force value set between 5 and 20 g. Following March of 2017, the ablation index (AI) was employed during the ablation procedure. The targeted AI values were set at 360–400 for the posterior and inferior walls, and 500–550 for the anterior and roof walls.

Following wide antral pulmonary vein isolation (PVI), physicians determined the need for supplemental ablation based on individual patient characteristics. In cases of PsAF, direct current cardioversion was administered to achieve sinus rhythm (SR) restoration. For instances of atrial tachycardia (AT) or atrial flutter (AFL), the underlying mechanism was first delineated through comprehensive activation and entrainment mapping prior to targeted ablation of critical isthmus regions or non-pulmonary vein foci. Upon successful SR restoration, all linear lesions underwent systematic evaluation to verify bidirectional conduction block. A standardized protocol involving intravenous isoprenaline infusion coupled with coronary sinus programmed electrical stimulation was implemented in all patients to provoke potential coexisting tachyarrhythmias.

### 2.5 Follow-up

For all patients, 24-h Holter monitoring was performed at 3, 6 and 12 months, followed by every 12 months. In case of palpitations or other related symptoms, a temporary 12-lead ECG or Holter monitoring was conducted to rule out the possibility of AF recurrence. The recurrence of AF was defined as any atrial tachyarrhythmia lasting more than 30 s, as evidenced by a 12-lead ECG or 24-h Holter monitoring after a blanking period of 3 months post-ablation, regardless of the number of procedures performed or the use of antiarrhythmic medication. Echocardiography was performed at 3, and 12 months, and subsequently on an annual basis after the ablation.

Antiarrhythmic drugs were prescribed for 1–3 months if necessary, and were discontinued after the 3-month blanking period, except for β-blockers. Proton pump inhibitors were administered for 4 weeks post-procedure. Oral anticoagulation was maintained for ≥3 months post-ablation, with long-term therapy determined by the patient's stroke risk score.

### 2.6 Statistical analysis

Continuous variables were expressed as mean ± standard deviation for normally distributed data or median (interquartile range) for non-normally distributed data, whereas categorical variables were presented as frequencies and percentages. For between-group comparisons, continuous variables were analyzed using Student's *t*-test (parametric) or Mann–Whitney *U*-test (non-parametric), as appropriate, while categorical variables were compared using the Chi-square test or Fisher's exact test when applicable. Multivariate Cox proportional-hazard models were utilized to identify the clinical predictors for AF recurrence. Variables with a *P*-value <0.10 in the univariate models were included in the multivariate analysis. Kaplan–Meier analysis was used to calculate atrial arrhythmia-free survival rates, and the log-rank test was employed to compare the rates among subgroups analysis. Logistic regression analysis was conducted to determine the risk factors for non-significant TR improvement after ablation. A *P*-value <0.05 was considered statistically significant. All statistical analyses were performed using SPSS for Windows (version 26, SPSS Inc., Chicago, IL, USA) and R software (version 4.3.1, R Studio).

## 3 Results

### 3.1 Patients' characteristics

The incidence of significant FTR was 9.1% (153/1,690). The study cohort comprised 306 patients with a mean age of 67.8 ± 10.3 years. AF duration exhibited a median of 24.0 months, accompanied by an average CHA2DS2-VASc score of 2.6 ± 1.2. Regarding TR severity, the cohort demonstrated a mean TR grade of 2.6 ± 1.0. The significant FTR included 88 patients with moderate FTR, 39 with moderate-to-severe FTR, and 26 with severe FTR. The details of patients' characteristics were shown in Table 1.

**Table 1:** Baseline characteristics of the study population and different severity of FTR groups.

Characteristics	Overall (*n* = 306)	No or mild FTR (*n* = 153)	Significant FTR (*n* = 153)	*P* *
Age, years	67.8 ± 10.3	68.0 ± 10.7	68.7 ± 10.6	0.141
Female gender, *n* (%)	150 (49.0)	65 (42.5)	85 (55.6)	0.022
BMI, kg/m^2^	24.6 ± 2.7	24.7 ± 2.9	24.5 ± 2.6	0.370
Significant MR, *n* (%)	102 (33.3)	48 (31.4)	54 (35.3)	0.467
AF duration, months	24.0 (12.0, 48.0)	21.0 (11.0, 36.0)	24.0 (12.0, 57.0)	0.002
Hypertension, *n* (%)	194 (63.4)	102 (66.7)	92 (60.1)	0.237
Dislipidemia, *n* (%)	80 (26.1)	41 (26.8)	39 (25.5)	0.796
Prior stroke/TIA, *n* (%)	58 (19.0)	31 (20.3)	27 (17.6)	0.561
Diabetes mellitus, *n* (%)	35 (11.4)	31 (20.3)	13 (8.5)	0.107
CAD, *n* (%)	35 (11.4)	16 (10.5）	19 (12.4)	0.591
Heart failure, *n* (%)	41 (13.4)	18 (11.8）	23 (15.0)	0.403
CHA_2_DS_2_-Vasc score	2.6 ± 1.2	2.6 ± 1.2	2.6 ± 1.3	0.854
Medications				
β-blocker, *n* (%)	183 (59.8)	92 (60.1)	91 (59.5)	0.908
Amiodarone, *n* (%)	25 (8.2)	12 (7.8)	12 (7.8)	0.835
Digoxin, *n* (%)	17 (5.6)	5 (3.3)	12 (7.8)	0.081
ACEI/ARB/ARNi, *n* (%)	164 (53.6)	78 (51.0)	86 (56.2)	0.359
LAD, mm	44.7 ± 5.3	42.8 ± 4.3	45.9 ± 5.5	<0.001
LADI, mm/m^2^	26.3 ± 3.7	25.3 ± 3.0	27.2 ± 4.0	<0.001
RA enlargement, *n* (%)	232 (75.8)	88 (57.5)	144 (94.1)	<0.001
RV enlargement, *n* (%)	70 (22.9)	14 (9.2)	56 (36.6)	<0.001
LVEF, %	60.6 ± 7.1	61.6 ± 5.7	59.6 ± 8.1	0.010
TR grade	2.6 ± 1.0	1.9 ± 0.8	3.2 ± 0.4	<0.001
PA pressure, mmHg	38.8 ± 11.0	34.5 ± 8.5	43.0 ± 11.6	<0.001
eGFR, ml/min/1.73 m^2^	77.6 ± 20.8	78.0 ± 21.9	77.2 ± 19.7	0.758
NT-proBNP, pg/ml	231.0 (147.0, 373.0)	202.0 (124.0, 349.0)	263.0 (168.5, 431.5)	0.005

ACEI, angiotensin-converting enzyme inhibitor; AF, atrial fibrillation; ARB, angiotensin receptor blocker; ARNi, angiotensin receptor/neprilysin inhibitor; BMI, body mass index; CAD, coronary artery disease; eGFR, estimated glomerular filtration rate; FTR, functional tricuspid regurgitation; MR, mitral regurgitation; TR, tricuspid regurgitation; LAD, left atrial diameter; LADI, left atrial diameter index; LVEF, left ventricular ejection fraction; NT-proBNP, N-terminal pro-B-type brain natriuretic peptide; PA, pulmonary artery; RA, right atrium; RV, right ventricle; TIA, transient ischemic attack.

* Comparison between patients with no or mild FTR and significant FTR.

**Figure 2:**
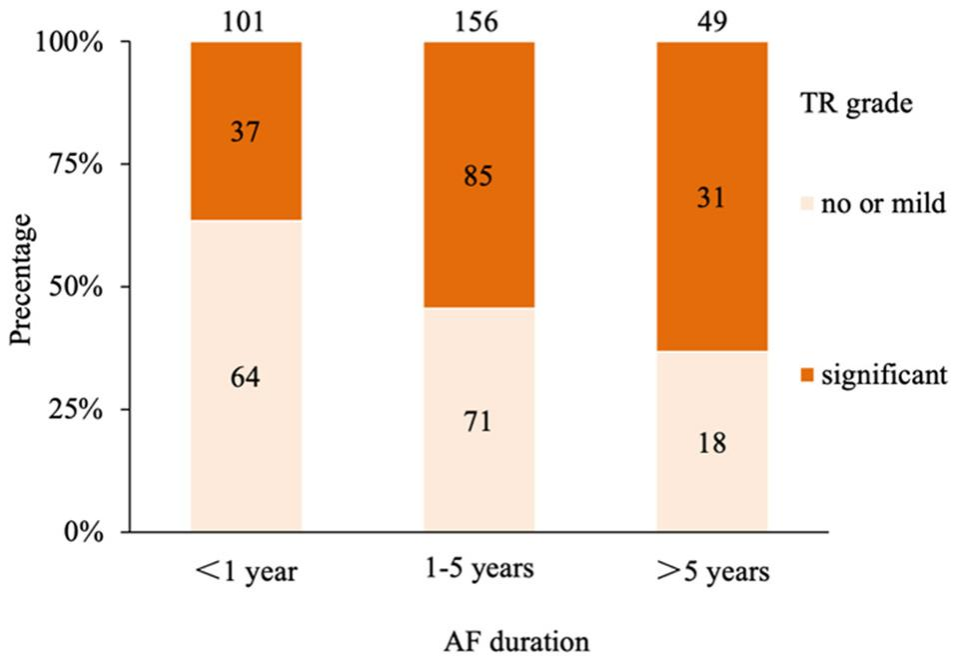
Distribution of grades of TR for different AF duration. TR, tricuspid regurgitation; AF, atrial fibrillation.

Significant FTR was less encountered in patients with AF duration less than 1 year, compared with those in whom AF duration was more than 1 year (37/101, 36.7% vs. 116/205, 56.6%, *P* < 0.001), and the distribution of significant FTR for patients with AF duration of 1–5 years and >5 years were 54.5% and 63.3%, respectively (Figure 2). Furthermore, significant differences were observed between patients with no or mild FTR and significant FTR in the proportion of female patients, AF duration, left atrial diameter (LAD), left atrial diameter index (LADI), right atrium (RA) enlargement, RV enlargement, pulmonary artery (PA) pressure, left ventricular ejection fraction (LVEF) and N-terminal pro-B-type natriuretic peptide (NT-proBNP) level. However, no significant differences were observed between the two groups in other baseline characteristics (Table 1).

Based on FTR subtypes, patients with vFTR (*n* = 70) showed significantly different clinical characteristics compared to those with no or aFTR (*n* = 236), including: longer AF duration, larger LAD, higher prevalence of heart failure, more frequent RA enlargement and amiodarone therapy, lower LVEF, higher LADI, more severe TR grade, elevated PA pressure, and increased NT-proBNP levels. No significant differences were observed between these two subgroups in other baseline characteristics (Table 2).

**Table 2:** Baseline characteristics of patients with No or aFTR and vFTR.

Characteristics	No or aFTR (*n* = 236)	vFTR (*n* = 70)	*P*
Age, years	67.3 ± 10.3	69.5 ± 10.2	0.130
Female gender, *n* (%)	124 (52.5)	32 (45.7)	0.317
BMI, kg/m^2^	24.7 ± 2.8	24.3 ± 2.6	0.392
Significant MR, *n* (%)	77 (32.6)	25 (35.7)	0.630
AF duration, months	21.0 (12.0, 36.0)	36.0 (21.75, 72.75)	<0.001
Hypertension, *n* (%)	150 (63.6)	44 (62.9)	0.915
Dislipidemia, *n* (%)	62 (26.3)	18 (25.7)	0.926
Prior stroke/TIA, *n* (%)	46 (19.5)	12 (17.1)	0.661
Diabetes mellitus, *n* (%)	30 (12.7)	5 (7.1)	0.200
CAD, *n* (%)	29 (12.3)	6 (8.6)	0.393
Heart failure, *n* (%)	26 (11.0）	15 (21.4)	0.025
CHA_2_DS_2_-Vasc score	2.6 ± 1.2	2.8 ± 1.2	0.268
Medications			
β-blocker, *n* (%)	134 (56.8)	49 (70.0)	0.048
Amiodarone, *n* (%)	14 (5.9)	11 (15.7)	0.009
Digoxin, *n* (%)	10 (4.2)	7 (10.0)	0.065
ACEI/ARB/ARNi, *n* (%)	124 (52.5)	40 (57.1)	0.498
LAD, mm	43.9 ± 5.0	46.2 ± 5.5	0.001
LADI, mm/m^2^	25.9 ± 3.7	27.4 ± 3.2	0.002
RA enlargement, *n* (%)	187 (79.2)	69 (98.6)	<0.001
LVEF, %	61.2 ± 6.2	58.5 ± 9.3	0.004
TR grade	2.3 ± 1.1	3.2 ± 0.5	<0.001
PA pressure, mmHg	37.4 ± 10.0	43.5 ± 13.0	<0.001
eGFR, ml/min/1.73 m^2^	77.6 ± 21.1	77.4 ± 20.0	0.946
NT-proBNP, pg/ml	220.0 (144.0, 351.0)	310.5 (194.0, 717.0)	<0.001

Abbreviations: see Table 1.

### 3.2 Procedural details

After PVI, roof linear ablation was performed in all patients, followed by inferoposterior line in 187 (61.1%) patients, mitral isthmus line in 96 (31.4%), and tricuspid isthmus line in 88 (28.8%). Superior vena cava isolation was performed in 32 (10.5%) patients. During the procedure, SR was restored by cardioversion in 175 (57.2%) patients, with a higher rate in patients with significant FTR than in those with no or mild FTR (105/153, 68.6%, vs. 70/153, 45.8%, *P* < 0.001). The mean procedure time was 251.9 ± 85.5 min, the mean ablation time was 78.8 ± 26.7 min, and the mean fluoroscopy time was 13.1 ± 8.9 min. There was no significant difference in procedural parameters between no or mild FTR group and significant FTR group. The procedural details are shown in Table 3.

**Table 3:** Procedural details of the study population and comparison between different severity of FTR groups.

Characteristics	Overall (*n* = 306)	No or mild FTR (*n* = 153)	Significant FTR (*n* = 153)	*P* *
Pulmonary vein isolation, *n* (%)	306 (100.0)	153 (100.0)	153 (100.0)	1.000
Linear ablation				
Roof line, *n* (%)	306 (100.0)	153 (100.0)	153 (100.0)	1.000
Inferoposterior line, *n* (%)	187 (61.1)	92 (60.1)	95 (62.1)	0.725
Mitral isthmus line, *n* (%)	96 (31.4)	46 (30.1)	50 (32.7)	0.622
Tricuspid isthmus line, *n* (%)	88 (28.8)	51 (33.3)	37 (24.2)	0.077
Isolation of superior vena cava, *n* (%)	32 (10.5)	15 (9.8)	18 (11.8)	0.580
Other additional ablation, *n* (%)	90 (29.4)	41 (26.8)	49 (32.0)	0.316
Cardioversion, *n* (%)	175 (57.2)	70 (45.8)	105 (68.6)	<0.001
Procedure time, min	251.9 ± 85.5	243.4 ± 72.3	260.5 ± 96.5	0.080
Ablation time, min	78.8 ± 26.7	76.1 ± 22.6	81.6 ± 30.0	0.069
Fluoroscopic time, min	13.1 ± 8.9	13.3 ± 8.3	12.5 ± 9.5	0.563

FTR, functional tricuspid regurgitation.

* Comparison between patients with no or mild FTR and significant FTR.

The overall procedural complication rate was 3.9% (12/306). Inguinal hematomas were observed in 3 patients with no or mild FTR and 5 patients with significant FTR. Pericardial tamponade developed in 3 cases: 1 patient in the no or mild FTR group and 2 patients in the significant FTR group (both resolved after pericardial drainage; 1 additional patient with significant FTR recovered spontaneously within 1 week post-procedure). A single case of cerebral infarction was noted on day 3 post-procedure in a patient with significant FTR.

### 3.3 Follow-up results

**Figure 3:**
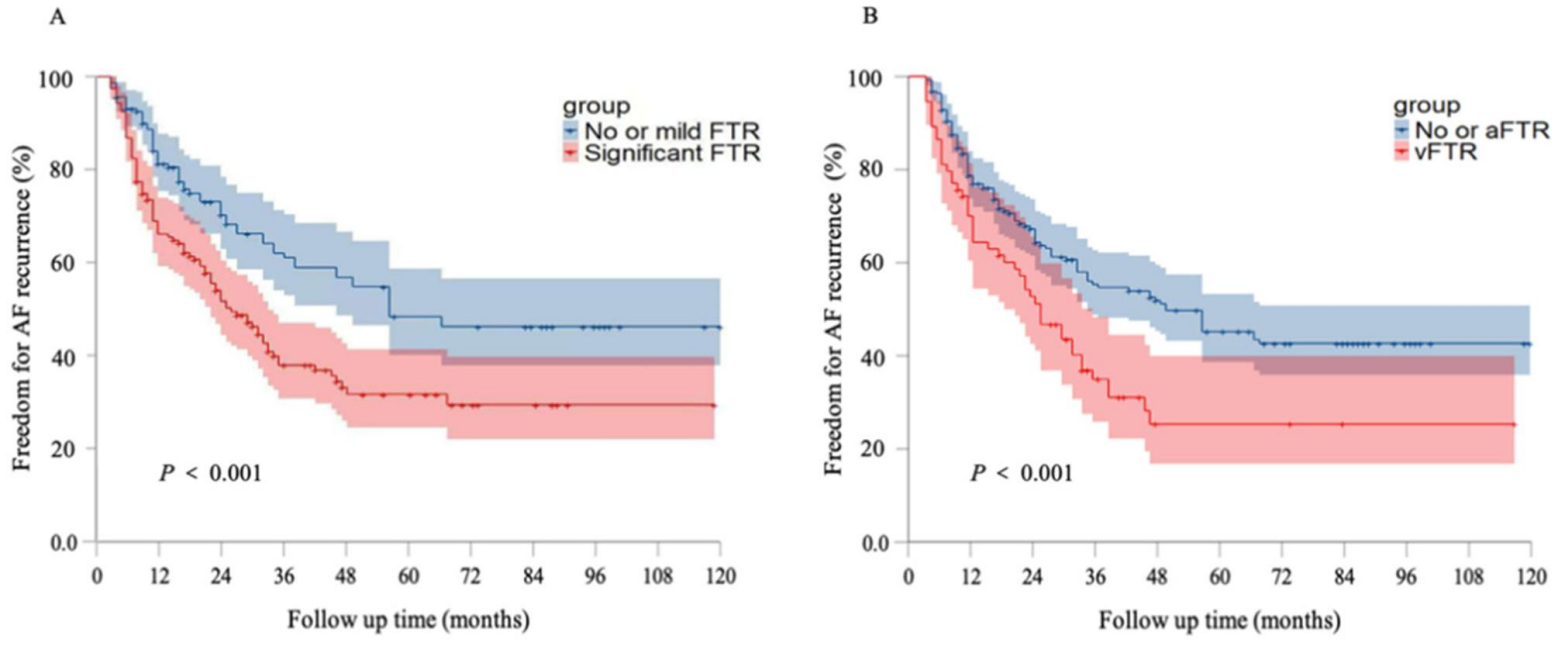
Kaplan–Meier survival curves of freedom for AF recurrence. **(A)** Kaplan–Meier survival curves of freedom for AF recurrence in different grades of FTR groups. **(B)** Kaplan–Meier survival curves freedom for AF recurrence in no or aFTR and vFTR groups. AF, atrial fibrillation; aFTR, atrial FTR; FTR, functional tricuspid regurgitation; vFTR, ventricular FTR.

After a mean follow-up period of 5.3 ± 3.7 years, AF recurred in 161 patients with an overall success rate of 47.4% (145/306) and significant difference was found in the overall success rate between no or mild FTR group and significant FTR group (57.5% vs. 37.3%, *P* < 0.001). The 12-month success rate was 67.6% (207/306), which was different between these 2 groups (75.8% vs. 59.5%, *P* = 0.002). The Kaplan–Meier curve of arrhythmia-free survival in different TR severity groups is shown in Figure 3A. Regarding to the subtype of FTR, the 12-month success rate was 71.2% (168/236) in patients with no or aFTR and 55.7% (39/70) in those with vFTR (*P* = 0.015), and there was significant difference in the overall success rate between patients with no or aFTR and those with vFTR (53.4% vs. 27.1%, *P* < 0.001). The Kaplan–Meier curve of arrhythmia-free survival in subtype of FTR is shown in Figure 3B.

During the follow-up period, a total of 6 patients died, including 3 patients with no or mild FTR and 3 patients with significant FTR. Cerebral infarction was encountered in another 2 patients in no or mild FTR group and 2 patients in significant FTR group. Two patients with recurrent atrial fibrillation in the significant FTR group underwent left atrial appendage occlusion during follow-up; one subsequently required permanent pacemaker implantation for sick sinus syndrome.

### 3.4 Predictors for AF recurrence

Univariable Cox regression analysis revealed that the AF duration, LAD, RA enlargement, RV enlargement, TR grade, PA pressure, eGFR, and NT-proBNP level were associated with AF recurrence. Subsequently, a multivariable Cox regression analysis was conducted to determine the independent predictors of AF recurrence after ablation. The analysis demonstrated that the AF duration [hazard ratio (HR) 1.008, 95% confidence interval (CI) 1.004–1.013, *P* < 0.001], TR grade (HR 2.379, 95% CI 1.403–4.036, *P* = 0.001), eGFR (HR 1.010, 95% CI 1.001–1.020, *P* = 0.033), and NT-proBNP level (HR 1.001, 95% CI 1.0002–1.002, *P* = 0.009) were significant predictors of AF recurrence after ablation.

### 3.5 FTR improvement after RFCA

**Figure 4:**
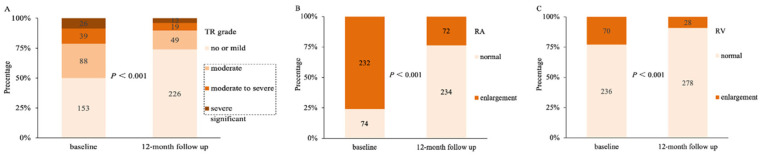
Changes of TR grade, RA enlargement and RV enlargement following RFCA. **(A)** Changes in the severity of TR from baseline to 12 months after RFCA. **(B)** Significantly improvement of RA enlargement from baseline to 12 months after RFCA. **(C)** Significantly improvement of RV enlargement from baseline to 12 months after RFCA. TR, tricuspid regurgitation; RA, right atrium; RV, right ventricle; RFCA, radiofrequency catheter ablation.

During the follow-up period of 12 months, RFCA was found to significantly improve the severity of FTR (Figure 4A), the percentage of significant TR after RFCA achieved 26.1% (80/306). Additionally, there was a significant reduction in the proportion of RA enlargement (232/306, 75.8% vs. 72/306, 23.5%, *P* < 0.001, Figure 4B) and RV enlargement (70/306, 22.9% vs. 28/306, 9.2%, *P* < 0.001, Figure 4C) after RFCA. Furthermore, there was a significant correlation between AF recurrence and FTR unimprovement from baseline to 12 months after RFCA. In the significant FTR group, 40.5% (62/153) of patients recurred, of which 58.7% (27/46) were FTR unimproved.

To identify risk factors of non-significant improvement in FTR after RFCA, logistic regression analyses were performed. AF duration [odds ratio (OR) 1.013, 95% CI 1.001–1.026, *P* = 0.039], RV enlargement (OR 4.167, 95% CI 1.746–10.550, *P* = 0.002), and AF recurrence (OR 18.244, 95% CI 7.500–52.427, *P* *<* 0.001) were identified as risk factors for non-significant improvement in FTR after ablation.

## 4 Discussion

### 4.1 Main finding

FTR in PsAF patients who underwent RFCA was not rare, which proved to be a significant predictor of AF recurrence during long-term follow-up. Particularly, subgroup analysis showed that the overall procedural success rate of patients with significant FTR and those with vFTR were significantly lower. RFCA demonstrated a significant improvement in FTR by reversal remodeling of RA and RV, and logistic regression analysis showed that AF recurrence was the strongest independent risk factor for non-significant improvement in FTR in PsAF patients following RFCA.

### 4.2 Characteristics of the study population

In the present study, among patients with PsAF undergoing RFCA, the incidence of significant FTR was 9.1%. Compared to earlier reports (8, 9, 13), differences in patient characteristics and grading of TR severity, could partly explain the inconsistencies in prevalence of FTR in these studies. However, The proportion of patients with significant FTR was 56.6% among those with atrial fibrillation persisting for more than 1 years, and nearly one-third of these patients had significant FMR in the study population. It was observed that a 75% dilatation of the mitral annulus was necessary to cause severe MR, whereas only a 40% dilatation of the tricuspid annulus was necessary to cause severe TR; compared to the mitral annulus, the tricuspid annulus has a different composition of myocardial and fibrous tissue, as well as variability in the papillary chordae tendinosus, making it more susceptible to dilatation in right heart remodeling (14). Our study suggested that the proportion of female in patients with significant FTR was higher compared to those with no or mild FTR. Females have significantly larger annulus circumferences and less elastic and cellular annuli compared to males, which may contribute to annular insufficiency and valvular incompetence (15). These sex differences in annulus structure provide a morphological basis for higher prevalence of significant FTR in female patients.

Consistent with previous research (3), we found that AF duration is strongly associated with severity of FTR, significant FTR was less encountered in patients with AF duration less than 1 year compared with those in whom AF duration was more than 1 year. Furthermore, it has been observed that larger LAD, RA/RV enlargement, higher LADI, PA pressure, NT-proBNP level, and lower LVEF are more commonly present in patients with significant FTR, indicating a potential contribution of FTR to the development of heart failure. AF is a progressive disease, with the persistence of AF and valvular regurgitation, atrial and ventricular remodeling, valvular regurgitation aggravation, and then followed by heart failure. A previous study showed significant FTR is associated with cardiac death and hospitalization due to worsening heart failure in patients with AF; moreover, the cumulative 1-year incidence of hospitalization for heart failure was very high in AF patients with FTR who did not undergo catheter ablation (3).

### 4.3 FTR and AF recurrence

Nakamura et al. (10) reported that AF recurrence was more frequent in patients with FTR compared to those without FTR (*P* = 0.001), and FTR was discovered as a predictive factor for AF recurrence after RFCA. In the present study, we found that AF recurrence was associated with the severity of FTR, during a long-term follow-up over 5 years, patients with significant FTR had a higher incidence of AF recurrence than those with no or mild FTR, and TR grade was found to be the most valuable predictor of AF recurrence after ablation. Nath et al. (16) demonstrated that mild or less TR did not affect outcomes, but moderate or greater TR was associated with worse outcomes, even in the absence of left ventricle dysfunction or PA hypertension. FTR is the result of the deformation of the TV annular, such as the dilatation and geometric deformation of the tricuspid annulus. FTR is also caused by increased afterload of the RV, which is associated with LA enlargement and PA hypertension. The severity of FTR is associated with RA/RV size, PA pressure and severity of FMR, which are also factors for AF recurrence, and the combination of FTR and FMR increases the risk of AF recurrence (10, 17–19).

FTR can be further divided into no or aFTR and vFTR, our study suggested that vFTR may result in a poorer prognosis than no or aFTR, the overall success rate in patients with no or aFTR was significantly higher than those with vFTR. Furthermore, vFTR was found to be associated with longer AF duration, larger LAD, higher proportion of heart failure, RA enlargement, lower LVEF, higher LADI, TR grade, PA pressure and NT-proBNP level. Florescu et al. (20) demonstrated that patients diagnosed with vFTR exhibited a more elliptic or spherical pattern of RV remodeling and a dysfunctional RV due to enlargement of mid diameters and lengths, while RV remodeling was less pronounced in patients with aFTR. aFTR and vFTR are more like a different stage of a process of TR in AF patients, as FTR worsens, there is progressive remodeling of the RV and RV dysfunction, which leads to further abnormalities in papillary muscle displacement and leaflet tethering, exacerbating FTR and RV dilatation. Schlotter et al. (21) found that patients with vFTR had more heart failure symptoms, more chronic obstructive pulmonary disease, and worse kidney function compared to those with aFTR, these factors are associated with AF recurrence after catheter ablation. Considering the underlying mechanisms in the development of FTR, it is critical to know that performing catheter ablation in the early stage of FTR was helpful in reducing AF recurrence.

### 4.4 RFCA and FTR improvement

Consistent with previous research (22–25), Our present study found that FTR severity can be significantly improved following RFCA through reversal remodeling of RA and RV. Markman et al. (23) demonstrated that in patients with moderate to severe FTR, the severity of FTR improved by at least one grade in 64% of AF patients following RFCA. Soulat-Dufour et al. (25) showed that the severity of FTR was improved after 12 months of follow-up in AF patients who were restored to SR through electrical cardioversion or RFCA. In the present study, AF recurrence was found to be the strongest independent risk factor for non-significant improvement in FTR in PsAF patients following RFCA. This result demonstrated a significant association between AF recurrence and non-significant improved FTR, the maintenance of SR plays a crucial role in the improvement of FTR. Additionally, the proportion of RA and RV enlargement significantly decreased after RFCA, the improvement of FTR observed in the present study was associated with reduction in RA and RV size. These findings support the notion that AF can lead to or exacerbate FTR through structural remodeling. However, further research would be needed to reveal the mechanisms of improvement of FTR after RFCA. Since it is still unclear whether the improvement of FTR is a result or cause of reverse remodeling of RA and RV. Compared to PVI or limited adjunctive line ablation, extensive ablation fails to confer significant improvements in FTR. Mohanty et al. (26) propose that extensive RFCA may induce left atrial stiff syndrome, thereby increasing atrial compliance load. This newly identified pathological mechanism may serve as a driver for TR progression. Furthermore, establishment of appropriate ablation strategies for PsAF patients with significant FTR is extremely important but challenging.

Risk factors for non-significant improved FTR also included RV enlargement. These findings suggest that structural remodeling is not completely reversible in PsAF patients with FTR, especially in patients with pre-existing structural abnormalities and those with very-long AF duration. However, early rhythm control is also important in PsAF patients with FTR, the earlier catheter ablation is performed, the better outcome may be achieved. As AF and TR are both progressive diseases, they tend to worsen each other, if left untreated, progression of diseases will affect patients' prognosis (8, 9). Different approaches such as surgical or transcatheter should be evaluated for AF patients at severe stages of TR to optimize outcomes and avoid repeated RFCA, if the estimated success rate of catheter ablation is extremely low (27).

## 5 Study limitations

This study has several limitations that need to be acknowledged. Firstly, it is a single-center retrospective study, which may lead to bias to results. Future prospective studies are recommended to confirm and improve upon these findings. Additionally, the follow-up time in this study was long, leading to a certain amount of missing data, which might have influenced the results. Furthermore, The ablation protocol appears to have evolved over time. While it introduces heterogeneity that could affect outcomes. Finally, temporary ECG and 24-h Holter were utilized for rhythm monitoring, this approach may have a lower detection rate for recurrence of asymptomatic AF. To overcome this limitation, alternative monitoring methods with higher sensitivity should be considered in future studies. Another limitation is that the severity of TR and MR were defined by the valve regurgitation jet area, which is a semi-quantitative measurement. Compared with quantitative measurements, this approach may introduce some inaccuracies in the evaluation of TR and MR severities.

## 6 Conclusions

FTR was an independent predictor of AF recurrence in patients with PsAF who underwent RFCA. The long-term success was poor in the subgroups of significant FTR and those with vFTR. Furthermore, RFCA appears to significantly improve severity of FTR by SR restoration. These results highlight the potential clinical significance of FTR in the management of AF.

## Data Availability

The original contributions presented in the study are included in the article/Supplementary Material, further inquiries can be directed to the corresponding authors.
